# Apoptosis, Mitochondrial Autophagy, Fission, and Fusion Maintain Mitochondrial Homeostasis in Mouse Liver Under Tail Suspension Conditions

**DOI:** 10.3390/ijms252011196

**Published:** 2024-10-18

**Authors:** Lu-Fan Li, Jiao Yu, Rui Li, Shan-Shan Li, Jun-Yao Huang, Ming-Di Wang, Li-Na Jiang, Jin-Hui Xu, Zhe Wang

**Affiliations:** School of Life Sciences, Qufu Normal University, Qufu 273165, China; 19553711591@163.com (L.-F.L.); 15954530736@163.com (J.Y.); 19553718390@163.com (R.L.); 19553714522@163.com (S.-S.L.); 17888370298@163.com (J.-Y.H.); d_1840644096@126.com (M.-D.W.); firstna@163.com (L.-N.J.)

**Keywords:** tail suspension, liver, mitochondria, apoptosis

## Abstract

Microgravity can induce alterations in liver morphology, structure, and function, with mitochondria playing an important role in these changes. Tail suspension (TS) is a well-established model for simulating the effects of microgravity on muscles and bones, but its impact on liver function remains unclear. In the current study, we explored the regulatory mechanisms of apoptosis, autophagy, fission, and fusion in maintaining liver mitochondrial homeostasis in mice subjected to TS for 2 or 4 weeks (TS2 and TS4). The results showed the following: (1) No significant differences were observed in nuclear ultrastructure or DNA fragmentation between the control and TS-treated groups. (2) No significant differences were detected in the mitochondrial area ratio among the three groups. (3) Cysteine aspartic acid-specific protease 3 (Caspase3) activity and the Bcl-2-associated X protein (bax)/B-cell lymphoma-2 (bcl2) ratio were not higher in the TS2 and TS4 groups compared to the control group. (4) dynamin-related protein 1 (DRP1) protein expression was increased, while mitochondrial fission factor (MFF) protein levels were decreased in the TS2 and TS4 groups compared to the control, suggesting stable mitochondrial fission. (5) No significant differences were observed in the optic atrophy 1 (OPA1), mitofusin 1 and 2 (MFN1 and MFN2) protein expression levels across the three groups. (6) Mitochondrial autophagy vesicles were present in the TS2 and TS4 groups, with a significant increase in Parkin phosphorylation corresponding to the duration of the TS treatment. (7) ATP synthase and citrate synthase activities were significantly elevated in the TS2 group compared to the control group but were significantly reduced in the TS4 group compared to the TS2 group. In summary, the coordinated regulation of apoptosis, mitochondrial fission and fusion, and particularly mitochondrial autophagy preserved mitochondrial morphology and contributed to the restoration of the activities of these two key mitochondrial enzymes, thereby maintaining liver mitochondrial homeostasis in mice under TS conditions.

## 1. Introduction

Prolonged exposure to microgravity can lead to significant physiological disruptions, including the redistribution of body fluids [1], dysregulation of the immune system [2], and oxidative stress in multiple organs and systems [3]. The liver, with its extensive blood supply and critical roles in synthesis and metabolism, is particularly susceptible to microgravity conditions [4]. Studies have shown that Mongolian gerbils (*Meriones unguiculatus*) experience an enlargement of hepatocyte nuclei and nucleoli following 12 days of spaceflight [5], while rats display a reduction in mitochondrial number beneath the soleus muscle membrane after 12–16 h in space [6]. Tail suspension (TS) is a well-established model for simulating the effects of microgravity on muscles and bones [7]. In male Sprague Dawley rats, 14 days of TS treatment accelerates mitochondrial degradation under the soleus muscle membrane compared to the degradation of mitochondria between myofibrils [8]. Mitochondria, the central sites of aerobic respiration and adenosine triphosphate (ATP) production, are essential for cellular energy metabolism. Previous research has shown that ATP synthase and citrate synthase (CS) activities are significantly increased in the mouse myocardium following 4 weeks of TS treatment [9]. However, it remains unclear whether TS affects the liver while impacting muscles and bones.

Mitochondrial function is intrinsically linked to mitochondrial homeostasis, which is maintained through a dynamic equilibrium of apoptosis, mitochondrial autophagy, fission, and fusion [10,11]. Apoptosis plays a critical role in clearing damaged cells and preserving normal tissue function [12]. We previously demonstrated that 4 weeks of TS treatment in mice results in a significant increase in apoptosis in cardiac myocytes, while maintaining stable apoptosis levels in the Harderian glands, with both effects linked to the ratio of Bcl-2-associated X protein (bax) to B-cell lymphoma-2 (bcl2) and cysteine aspartic acid-specific protease 3 (Caspase3) activity [9,13]. However, the effect of the mitochondria-mediated apoptotic pathway on the liver under TS conditions remains unresolved.

Mitochondrial autophagy is essential for mitochondrial homeostasis through the degradation of dysfunctional mitochondria [11,14,15]. Parkinson disease protein 2 (Parkin), a key regulator of mitochondrial autophagy, becomes activated through phosphorylation, transitioning into active phosphorylated Parkin (P-Parkin) [16]. Prior studies have shown that the Parkin phosphorylation ratio is elevated in the Harderian glands and heart after 4 weeks of TS treatment compared to 2 weeks, indicating heightened autophagic activity [9,13]. This suggests that Parkin may play a critical role in mediating the effects of TS on mitochondrial autophagy in the livers of mice.

Mitochondrial fission and fusion, functioning in conjunction with mitochondrial autophagy, are critical processes that regulate mitochondrial homeostasis [17]. Previous studies have reported that 4 weeks of TS treatment results in a reduction in the levels of mitofusin 1 and 2 (MFN1 and MFN2), as well as dynamin-related protein 1 (DRP1) in the gastrocnemius and tibialis anterior muscles of mice, suggesting that both mitochondrial fission and fusion are inhibited [18]. Thus, investigating these key regulators of fission and fusion should provide further insights into the influence of TS on liver mitochondrial homeostasis in mice.

The TS animal model primarily affects two aspects of the body, namely muscle atrophy due to hind limb disuse and alterations in systemic blood flow distribution. As the liver is highly sinusoidal, its response to such hemodynamic changes remains unclear. In this study, we hypothesize that TS will induce changes in mitochondrial homeostasis in the liver of mice, driven by the regulation of mitochondrial autophagy, fission, fusion, and apoptosis, depending on the duration of exposure. Thus, we subjected mice to TS for varying durations (control group, 2-week group, and 4-week group) and performed quantitative analyses of the key factors involved in the pathways related to apoptosis, mitochondrial dynamics, and the activities of two key mitochondrial enzymes, with the aim of uncovering the cellular and molecular mechanisms underlying these changes.

## 2. Results

### 2.1. Influence of TS on Body Weight (BW), Body Length (BL), Carcass Weight (CW), Liver Weight (LW), LW-to-BW Ratio (LW/BW), and LW-to-CW Ratio (LW/CW) in Mice

**Figure 1:**
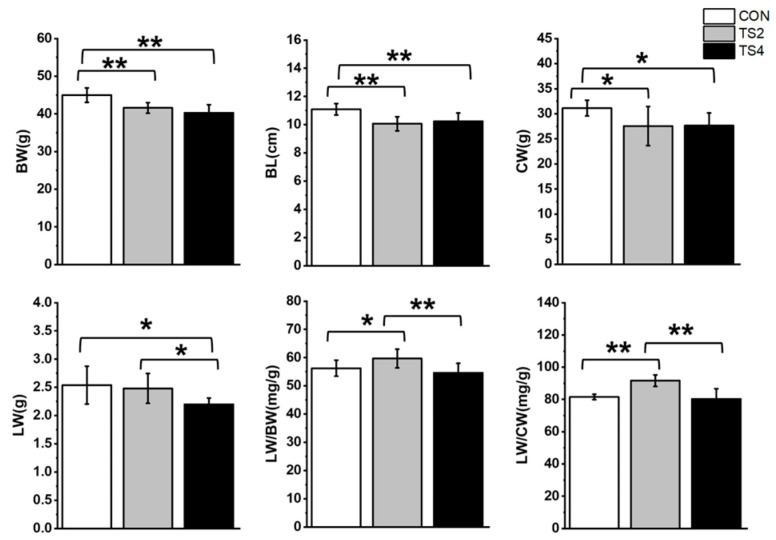
Influence of TS on morphological data in mice. Numerical values are mean ± standard deviation. n = 8. CON, control group; TS2, tail suspension 2-week group; and TS4, tail suspension 4-week group. ** *p* < 0.01 and * *p* < 0.05.

The BW, BL, and CW of the TS2 and TS4 groups were significantly lower than those of the control (CON) group (*p* < 0.05), with no significant differences observed between the TS2 and TS4 groups. The TS4 group exhibited the lowest LW (*p* < 0.05), while no significant difference in LW was found between the CON and TS2 groups. The TS2 group showed the highest LW/BW and LW/CW ratios (*p* < 0.05), while no significant differences in these ratios were detected between the CON and TS4 groups (Figure 1).

### 2.2. Hematoxylin and Eosin (H&E) Staining of Liver Tissue

**Figure 2:**
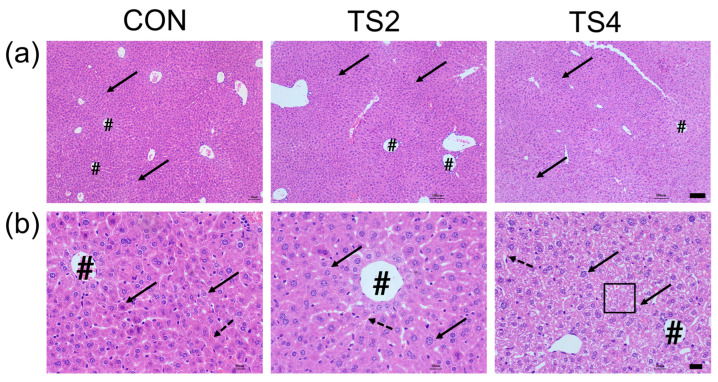
Histological morphology of mouse liver under different TS treatments. (**a**) Scale = 100 μm. (**b**) Scale = 20 μm. Arrows point to hepatocytes. Dashed arrows point to sinusoids. # shows vasculature. Cells within square boxes are necrotic liver cells. CON, control group; TS2, tail suspension 2-week group; and TS4, tail suspension 4-week group.

In the H&E staining results, hepatocyte nuclei appeared blue-violet, with some cells exhibiting binucleation. The hepatocytes were arranged in a radial pattern around the central vein, with sinusoids present between them. In the CON and TS2 groups, hepatocytes showed a close and orderly arrangement, with intact hepatic lobule structures. However, in the TS4 group, localized necrosis of hepatocytes and dissolution of cell nuclei were evident (Figure 2).

### 2.3. Ultrastructural Changes in Liver Mitochondrial and Nuclei

**Figure 3:**
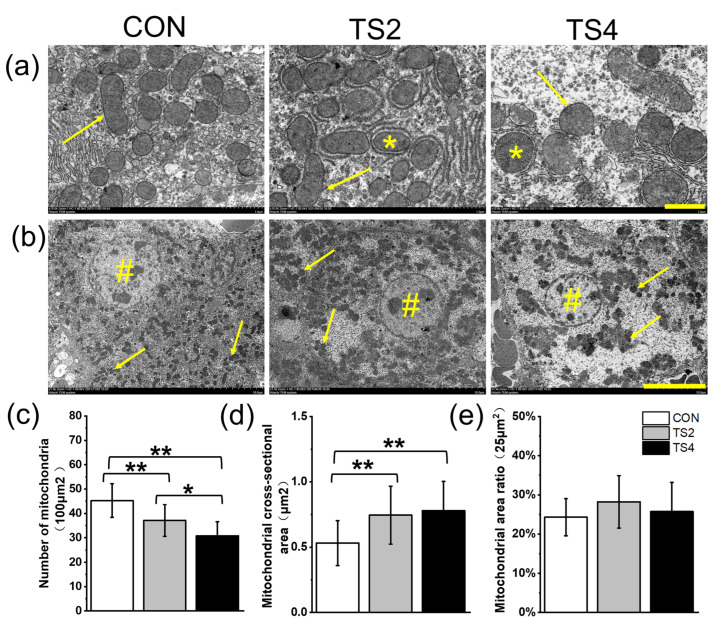
Ultrastructure of mouse liver tissue and analysis of mitochondrial number and area under different TS treatments. (**a**) Scale = 1 μm. * shows mitochondrial autophagic vesicles. Arrows point to mitochondria. (**b**) Scale = 10 μm. # shows liver nucleus. Arrows point to mitochondria. (**c**) Number of mitochondria. (**d**) Mitochondrial cross-sectional area. (**e**) Mitochondrial area ratio. Numerical values are mean ± standard deviation. Fifteen pictures were analyzed in each group. CON, control group; TS2, tail suspension 2-week group; and TS4, tail suspension 4-week group. ** *p* < 0.01 and * *p* < 0.05.

The ultrastructural examination of liver tissue sections and the analysis of mitochondrial number and area are shown in Figure 3. The mitochondria in the liver tissues of the three experimental groups showed minimal signs of cristae fracture and vacuolization. Mitochondrial autophagic vesicles were observed in the TS groups (Figure 3a), although no chromatin agglutination or nuclear damage was observed (Figure 3b). In the same region, the number of mitochondria decreased significantly with the increase in TS duration (*p* < 0.05, Figure 3c). The cross-sectional area of liver mitochondria in the TS groups was significantly larger in the CON group (*p* < 0.05), although there was no significant difference between the two TS groups (Figure 3d). The ratio of mitochondrial area to total area remained stable across all three groups (Figure 3e).

### 2.4. Variations in ATP Synthase, CS, and Caspase3 Activity

**Figure 4:**
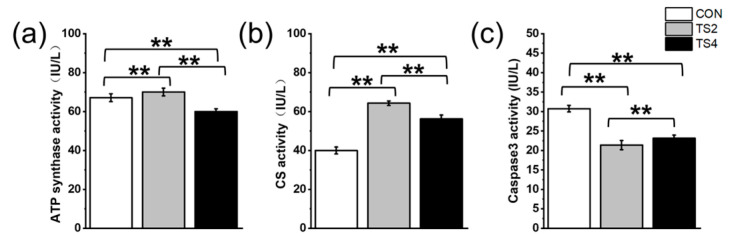
Activities of ATP synthase (**a**), CS (**b**), and Caspase3 (**c**) in mouse liver under different TS treatments. Numerical values are mean ± standard deviation. n = 8. CON, control group; TS2, tail suspension 2-week group; and TS4, tail suspension 4-week group. ** *p* < 0.01.

ATP synthase plays a crucial role in cellular oxidative phosphorylation, while CS is a key enzyme in the tricarboxylic acid (TCA) cycle. The results showed that ATP synthase activity was the highest in the TS2 group and the lowest in the TS4 group, showing an initial increase followed by a decrease with increasing TS duration (*p* < 0.05, Figure 4a). Similarly, CS activity was significantly higher in the TS groups compared to the CON group, and significantly higher in the TS2 group compared to the TS4 group, showing an initial increasing trend followed by a decreasing trend with increasing TS duration (*p* < 0.05, Figure 4b). In contrast, Caspase3 activity was significantly lower in the TS groups compared to the CON group, with the lowest activity observed in the TS2 group (*p* < 0.05, Figure 4c).

### 2.5. DNA Fragmentation

**Figure 5:**
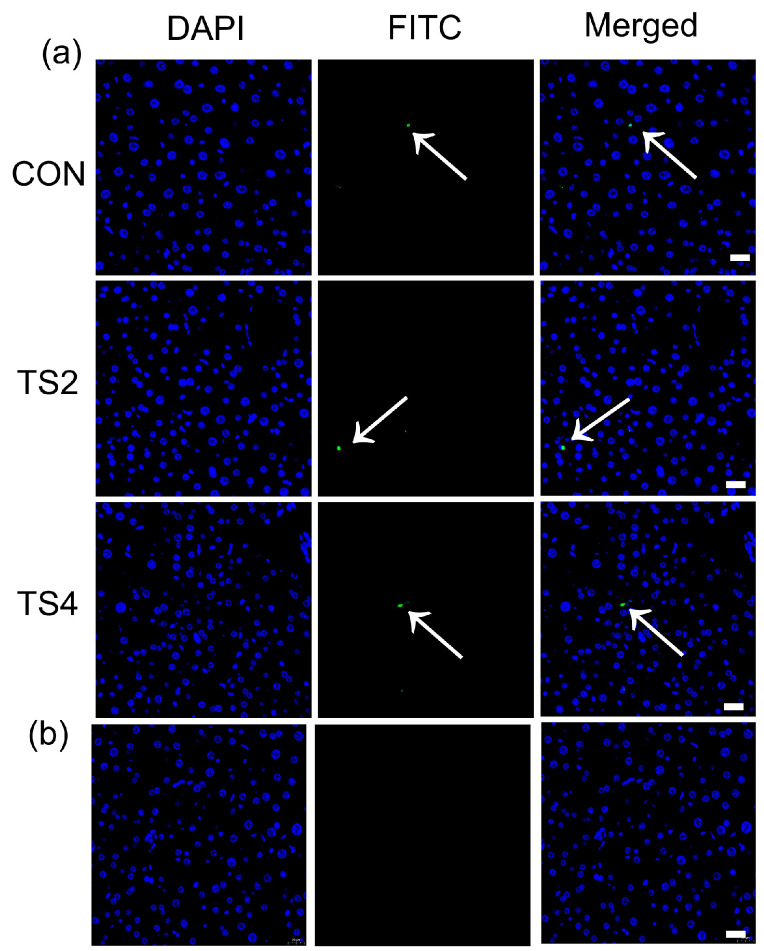
TUNEL staining of mouse liver under different TS treatments. (**a**) Scale = 20 μm. Arrows point to DNA fragmentation. Blue fluorescence indicates nuclei; green fluorescence indicates DNA fragmentation. (**b**) Negative control for TUNEL staining of mouse liver. Scale = 20 μm. CON, control group; TS2, tail suspension 2-week group; and TS4, tail suspension 4-week group.

TUNEL staining was employed to detect apoptosis in liver tissue based on DNA fragmentation. Figure 5a,b illustrate the TUNEL staining results for the CON, TS2, and TS4 groups, as well as the negative control, with blue fluorescence indicating the location of nuclei and green fluorescence indicating areas of DNA fragmentation. As shown in Figure 5a, DNA fragmentation was infrequent and limited across all three groups, indicating minimal apoptotic activity (Figure 5a).

### 2.6. Variations in Expression of Apoptosis-Associated Proteins

**Figure 6:**
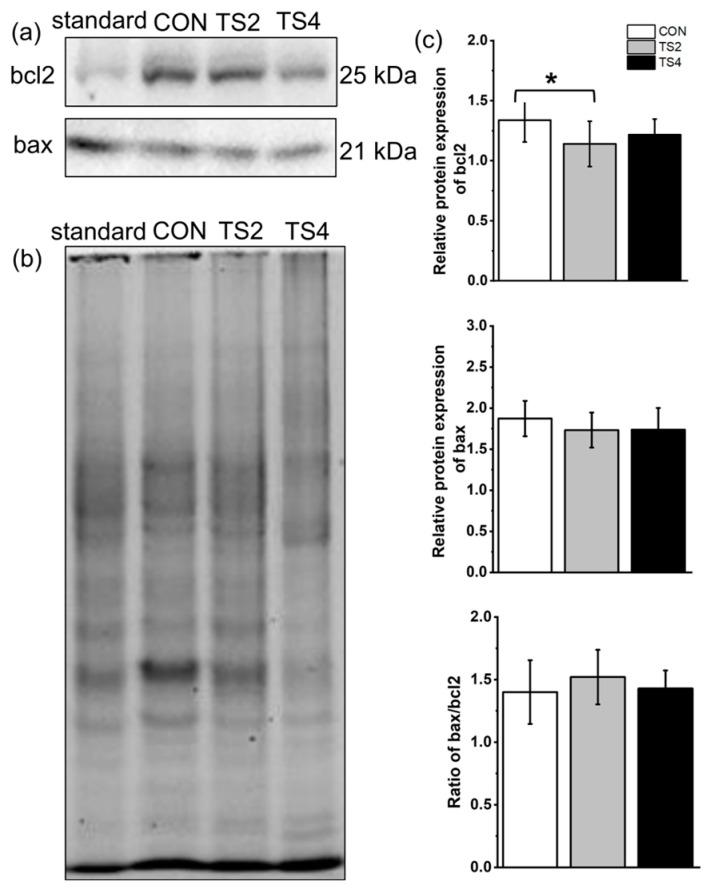
Expression levels of liver apoptosis-associated proteins in mice under different TS treatments. (**a**) Representative Western blot gels. (**b**) Polyacrylamide gel of total protein. (**c**) Apoptosis-associated protein level. Numerical values are mean ± standard deviation. n = 8. CON, control group; TS2, tail suspension 2-week group; and TS4, tail suspension 4-week group. * *p* < 0.05.

Western blot analysis was performed to assess the expression of apoptosis-related proteins, with representative gels for bcl2 and bax shown in Figure 6a and the total protein gel displayed in Figure 6b. The relative expression level of bcl2 was highest in the CON group and lowest in the TS2 group (*p* < 0.05). No significant differences were found in the relative protein expression of bax among the three groups. The bax/bcl2 ratio did not differ significantly among the three groups (*p* < 0.05, Figure 6c).

### 2.7. Variations in Expression of Mitochondrial Fission- and Fusion-Associated Proteins

**Figure 7:**
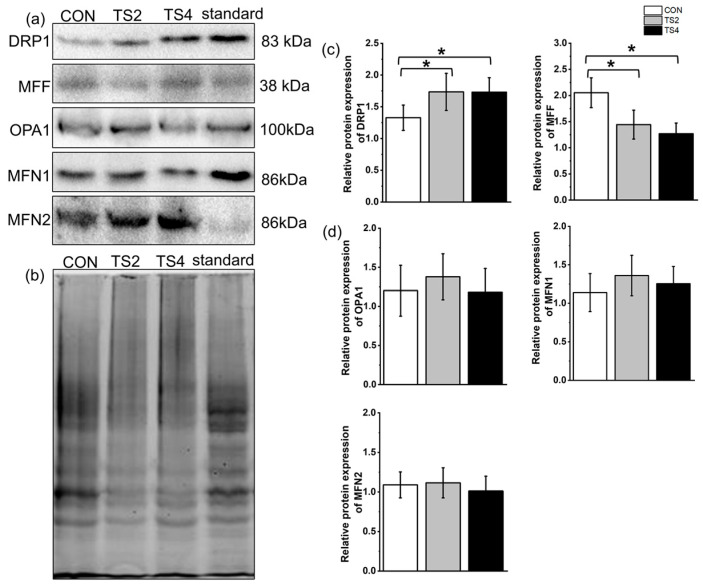
Expression levels of mitochondrial fission- and fusion-associated proteins in mice liver mitochondria under different TS treatments. (**a**) Representative Western blot gels. (**b**) Polyacrylamide gel of total protein in the liver. (**c**) Mitochondrial fission-associated protein levels. (**d**) Expression levels of mitochondrial fusion-associated proteins. Numerical values are mean ± standard deviation. n = 8. CON, control group; TS2, tail suspension 2-week group; and TS4, tail suspension 4-week group. * *p* < 0.05.

Representative Western blot gels of DRP1, mitochondrial fission factor (MFF), optic atrophy 1 (OPA1), MFN1, and MFN2 are shown in Figure 7a, with the total protein gel displayed in Figure 7b. Compared to the CON group, the protein expression of DRP1 was significantly increased in the TS2 and TS4 groups, while MFF expression was significantly decreased (*p* < 0.05). However, no significant differences were observed in the relative protein expression of DRP1 and MFF between the two TS groups (Figure 7c). Additionally, no significant differences in the protein expression levels of OPA1, MFN1, and MFN2 were detected among the three groups (Figure 7d).

### 2.8. Variations in Expression of Mitochondrial Autophagy-Associated Proteins

**Figure 8:**
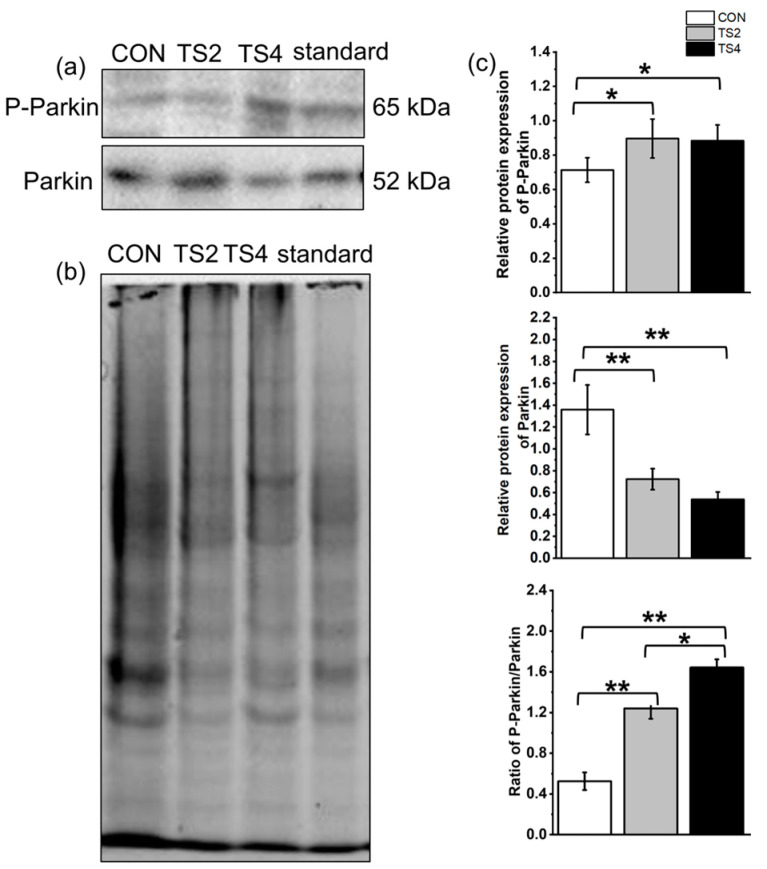
Expression levels of autophagy-associated proteins in mouse liver mitochondria under different TS treatments. (**a**) Representative Western blot gels. (**b**) Polyacrylamide gel of total protein. (**c**) Mitochondrial autophagy-associated protein levels. Numerical values are mean ± standard deviation. n = 8. ** *p* < 0.01 and * *p* < 0.05.

Representative Western blot gels of Parkin and P-Parkin are shown in Figure 8a, with the total protein gel depicted in Figure 8b. Compared to the CON group, the relative expression of P-Parkin increased significantly in the TS2 and TS4 groups, while the relative expression of Parkin decreased significantly. Additionally, the P-Parkin/Parkin ratio was reduced in the TS groups (*p* < 0.05, Figure 8c).

## 3. Discussion

**Figure 9:**
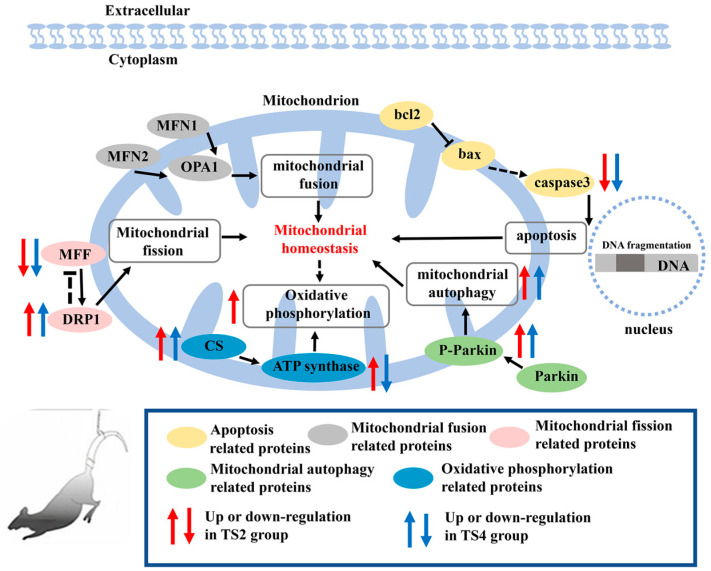
Summary of impact of TS on liver mitochondrial homeostasis in mice. bcl2, B-cell lymphoma-2; bax, Bcl-2-associated X protein; caspase3, cysteine aspartic acid-specific protease 3; MFN1, mitofusin 1; MFN2, mitofusin 2; OPA1, optic atrophy 1; MFF, mitochondrial fission factor; DRP1, dynamin-related protein 1; Parkin, Parkinson disease protein 2; P-Parkin, phosphorylated Parkin; ATP synthase, adenosine triphosphate synthase; and CS, citrate synthase. Yellow represents apoptosis-related proteins. Gray represents mitochondrial fusion-related proteins. Pink represents mitochondrial fission-related proteins. Green represents mitochondrial autophagy-related proteins. Blue represents oxidative phosphorylation-related proteins. Red arrows represent up- or down-regulation in TS2 group. Blue arrows represent up- or down-regulation in TS4 group.

This study investigated the impact of TS on liver mitochondrial homeostasis in mice, focusing on the potential regulatory mechanisms involving apoptosis, mitochondrial autophagy, fission, and fusion (Figure 9).

The results demonstrated that TS treatment significantly reduced BL in mice, indicating an inhibition of growth. Additionally, both BW and LW decreased following TS, consistent with previous research [19,20,21]. However, the LW/BW ratio in the TS2 group was higher, possibly due to a greater reduction in BW relative to LW, suggesting that liver maintenance may be prioritized over other organs under TS conditions.

Ultrastructural observations revealed no evidence of chromatin agglutination or nuclear damage in hepatocyte nuclei. The reduced activity of Caspase3, a key protease promoting apoptosis [22], along with the unchanged bax/bcl2 ratio, an upstream regulator of Caspase3 [23], suggests that cell apoptosis did not increase in either TS group. This is consistent with studies showing that altering gravity conditions via parabolic flight maneuvers does not affect apoptosis levels in ML-1 thyroid cancer cells [24] or induce apoptosis in endothelial cells [25]. Additionally, our findings align with previous research demonstrating no mitochondrial damage in PICM-19 porcine liver stem cells after 16 days of space culture [26], suggesting preserved mitochondrial integrity in the liver and stable apoptosis levels. Our prior research indicated that mitochondria in the mouse heart displayed swelling and disrupted cristae after two weeks of TS treatment [9], implying that mitochondrial structure in the liver may exhibit greater resilience under similar conditions. In contrast, other studies have reported hepatocyte apoptosis in rats following 7-day and 2-month TS treatments [27,28]. The variation in hepatocyte apoptosis between mice and rats may be related to species-specific differences, with the hepatocytes of mice potentially being less susceptible to apoptosis than those in rats [29].

DRP1, a protein that accumulates on the outer mitochondrial membrane to induce fission [30,31,32,33], was up-regulated in the TS2 and TS4 groups, while MFF, a key receptor for recruiting and activating DRP1 [34,35,36], was down-regulated. These changes suggest that mitochondrial fission in the liver remained balanced despite TS exposure, consistent with our previous findings showing stable mitochondrial fission levels in the myocardia across the three groups [9]. Additionally, the expression of MFN1, MFN2, and OPA1, proteins critical for maintaining mitochondrial fusion [32,33,34,35], was stable across all groups, suggesting that TS did not significantly affect mitochondrial fusion in the liver. This contrasts with our earlier findings in the mouse heart, where TS was associated with an increase in cardiac mitochondrial fusion [9], highlighting potential organ-specific responses to TS conditions.

A key finding of this study is that mitochondrial autophagy increased with TS duration, which likely accounts for the observed reduction in mitochondrial numbers. The presence of mitochondrial autophagic vesicles and the elevation of Parkin phosphorylation in liver cells of TS-treated mice indicated an up-regulation in the level of mitochondrial autophagy. Previous studies have demonstrated that simulated microgravity can alter immune function and induce cellular stress responses in various tissues [37,38]. These immune- and stress-related changes often influence mitochondrial autophagy, facilitating the clearance of damaged mitochondria and promoting self-renewal through enhanced autophagic activity [39,40]. Microgravity can activate metabolic pathways, leading to increased production of reactive oxygen species (ROS) [41], while prolonged TS conditions can cause uneven fluid distribution [42], both of which may contribute to oxidative stress in multiple organs [43], driving mitochondrial fragmentation and hepatocyte apoptosis [44,45,46]. However, no evidence of mitochondrial fragmentation was observed, and the mitochondrial area ratio remained stable. Apoptosis levels across the three groups also showed no significant differences, likely due to increased mitochondrial autophagy, which effectively cleared damaged mitochondria and alleviated the oxidative stress response in hepatocytes [47]. Additionally, mitochondrial autophagy ensures that only healthy mitochondria engage in fission and fusion [48], thereby maintaining mitochondrial homeostasis.

In our study, mitochondrial homeostasis in the liver of TS-treated mice remained well preserved. We speculate that this may be due to adaptive changes in the liver occurring earlier than those in the heart, as degenerative changes in cardiac mitochondria were observed after 2 weeks of TS in mice [9]. Alternatively, the impact of TS on the liver may be less severe compared to the heart, resulting in significant changes in the heart after 2 weeks of TS, while the liver remains stable. Studies comparing the effects of real microgravity and TS on the liver have shown elevated levels of liver injury markers, alanine aminotransferase (ALT), and aspartate aminotransferase (AST), in the blood of rats following 18.5 days of spaceflight and 6 weeks of TS treatment [4,49]. However, hepatic glycogen content in male rats exhibited opposite trends after 14 days of spaceflight and 2 months of TS treatment [28,50]. Therefore, whether TS can fully replicate the hepatic homeostasis changes observed in space may only be conclusively determined through experiments conducted under the actual microgravity environment of space.

ATP synthase and citrate synthase (CS) activities in the livers of mice were significantly elevated in the TS2 group compared to the CON group but significantly reduced in the TS4 group relative to the TS2 group. Research utilizing the Random Positioning Machine (RPM) system to simulate microgravity in human osteoblasts has demonstrated weakened mitochondrial respiratory chain function and reduced ATP synthesis capability in these cells [51]. However, studies have shown that the RPM system cannot fully converge the mean gravity to zero over time [52]. Given that osteoblasts are highly sensitive to mechanical forces, and while our findings are similar, further validation is needed to determine whether the reduced ATP synthesis in osteoblasts is indeed a consequence of microgravity exposure. Skeletal muscle and the liver are key thermogenic organs in the body. Previous studies have shown that TS treatment for 3 days increases ATP content in rat soleus muscles [53,54], while 7 days of treatment decreases CS activity in the gastrocnemius muscles of mice [55], 21 days of treatment reduces CS activity in rat soleus muscles [56], and prolonged treatment induces varying degrees of muscle atrophy in rat gastrocnemius and soleus muscles [57]. These findings suggest that short-term TS treatment may enhance ATP production in skeletal muscles, while long-term TS treatment may weaken mitochondrial function in these muscles. The trends in mitochondrial enzyme activity changes observed in our study are consistent with these patterns in skeletal muscles under TS treatment. Research has shown that oxidative respiration in the liver can return to normal physiological levels after 21 days of TS [58]. Thus, in contrast to the functional decline seen in skeletal muscles under prolonged TS, the liver appears to have a stronger adaptive response to the TS state. This adaptability is likely associated with the dynamic regulation of mitochondrial autophagy, fission, and fusion [59], with increased mitochondrial autophagy playing a key role. Our previous research also demonstrated adaptive changes in the heart in response to prolonged TS, with elevated levels of mitochondrial autophagy and fusion contributing to an increase in the number of mitochondria and the maintenance of their morphology in the heart after 4 weeks of TS treatment [9]. Compared to the TS2 group, the lower ATP synthase and CS activities in the TS4 group may suggest a return to normal physiological conditions or a reduction in the metabolic changes observed in the TS2 group. Previous research has shown that oxidative respiration in the liver can return to normal physiological levels after 21 days of TS [58], indicating that long-term TS treatment can lead to adaptability in mice. This adaptability is likely associated with the dynamic regulation of mitochondrial autophagy, fission, and fusion [59], with increased mitochondrial autophagy playing a key role. Similar findings have been reported in studies simulating microgravity in human osteoblasts, where altered centrifugal forces result in decreased mitochondrial respiratory function and reduced ATP synthesis capacity [51]. Mitochondrial autophagy plays a crucial role in regulating mitochondrial stress [60,61]. Under increased TS duration, up-regulation of mitochondrial autophagy may help alleviate ATP synthase and CS hyperactivity, thus supporting mouse liver adaptation to the TS state.

In conclusion, this study explored the regulatory mechanisms of apoptosis, mitochondrial autophagy, fission, and fusion in maintaining mitochondrial morphology, quantity, and function in the livers of mice subjected to different durations of TS. The results showed that apoptosis, mitochondrial fission, and mitochondrial fusion remained stable across all groups, while mitochondrial autophagy increased with TS duration. This up-regulation of autophagy may help alleviate the over-activity of ATP synthase and CS activity induced by TS, contributing to the stability of mitochondrial apoptosis, fission, and fusion. Overall, the dynamic regulation of apoptosis, mitochondrial fission, and fusion, particularly enhanced autophagy, played a critical role in preserving mitochondrial morphology and facilitated the recovery of the activities of two mitochondrial functional enzymes, enabling the maintenance of mitochondrial homeostasis in the livers of mice under TS conditions.

## 4. Materials and Methods

### 4.1. Ethics Statement

Statement on the welfare of animals. All procedures followed the Laboratory Animal Guidelines for the Ethical Review of Animal Welfare (GB/T 35892-2018) [62] and were approved by the Biomedical Ethics Committee of Qufu Normal University (Permit Number: dwsc2024111).

### 4.2. Animals and Groups

Kunming (KM) mice (*Mus musculus*) were purchased from Pengyue Experimental Animal Breeding Co., Ltd. (Jinan, China). Following a two-week period of adaptation to the laboratory with free movement, the mice were weighed, numbered, and randomly assigned to one of the three groups (n = 8 in each). The mice were all 7 weeks old at the beginning of the formal experiment. The three groups included the following: a control group (CON) in which mice were allowed to move freely for 4 weeks; the 2-week TS group (TS2) in which mice after 2 weeks of free movement were subjected to tail suspension for 2 weeks, during which they were suspended at an angle of 30° between their body and the horizontal plane; and the 4-week TS group (TS4) in which mice were subjected to tail suspension for 4 weeks during which they were suspended at an angle of 30° between their body and the horizontal plane [63,64]. For all three groups, the same conditions of housing were maintained, such as light (12:12 h light/dark and light on from 06:00 to 18:00), temperature (22 ± 2 °C), and humidity (55 ± 5%), and they were all 11 weeks old after receiving 4 weeks of treatment.

### 4.3. Sample Preparation

After 4 weeks of treatment, all animals were sacrificed via CO_2_ asphyxiation between the hours of 08:00 and 12:00 [9]. The liver was then quickly removed and weighed. The right anterior lobe of the liver from each group was frozen with liquid nitrogen and stored at −80 °C for Western blot and protein activity analysis. The remaining liver was used for transmission electron microscopy and frozen section experiments. All procedures were carried out in accordance with the approved guidelines.

### 4.4. Hematoxylin and Eosin (H&E) Staining

The liver was sectioned into 6 μm tissue slides after paraffin embedding and mounted on glass slides. Following gradient dehydration, the slices were stained with hematoxylin (30 min) and eosin (2 min), then dehydrated and sealed to create permanent slides [65]. An optical microscope (Olympus, BX51, Tokyo, Japan) was used to observe liver morphology.

### 4.5. Transmission Electron Microscopy (TEM)

Glutaraldehyde-preserved livers were dehydrated in a series of ethanol solutions at varying concentrations. The samples were embedded in epoxy resin [66] and precisely cut into ultrathin sections using an ultra-microtome. The sections were subjected to dual staining with Reynolds’ lead citrate and ethanoic uranyl acetate [67] before being observed and photographed using a TEM (Hitachi, HT7800, Tokyo, Japan).

### 4.6. ATP Synthase, CS, and Caspase3 Activity

Samples stored at −80 °C were used to detect ATP synthase, CS, and Caspase3 activity. ATP synthase activity was determined by measuring the free phosphate group at 660 nm using a Mitochondrial Complex V/ATP synthase Activity Assay Kit (BC1445, Solarbio, Beijing, China) according to the manufacturer’s instructions [68]. The CS activity was determined by measuring coenzyme A (CoA) formation at 450 nm with a Citrate Synthase Activity Assay Kit (BC1060, Solarbio) according to the manufacturer’s instructions [69]. Here, Caspase3 activity in the cell lysates was determined using a Caspase 3 Activity Kit (BC3830, Solarbio) following the manufacturer’s protocols [70].

### 4.7. Terminal Deoxynucleotidyl Transferase Biotin-dUTP Nick End Labeling (TUNEL) Staining

Paraffin sections designated for TUNEL staining were dewaxed and subsequently stained using a TUNEL Apoptosis Assay Kit (#MK1013, Boster, Wuhan, China) in accordance with the provided instructions and following previously reported procedures [65].

### 4.8. Western Blotting

Frozen tissue samples were homogenized in a RIPA lysis buffer. Soluble protein concentration was determined using a Pierce™ BCA Protein Quantification Kit (#53225, Thermo Fisher Scientific, Waltham, MA, USA). The samples were then adjusted to a final protein concentration of 3 μg/μL with 1 × SDS loading buffer (#1112, Boster, Wuhan, China) and a homogenizing buffer. The final protein samples were stored at −80 °C until further use. The primary antibodies used for targeting specific proteins were bax (1:1000, #50599, Proteintech, Wuhan, China), bcl2 (1:1000, #3498, Cell Signaling Technology, Danvers, MA, USA), DRP1 (1:2000, #12957, Proteintech, Wuhan, China), MFF (1:5000, #17090, Proteintech), OPA1 (1:2000, #27733, Proteintech), MFN1 (1:1000, #13798, Proteintech), MFN2 (1:2000, #12186, Proteintech), Parkin (1:1000, #14060, Proteintech), and P-Parkin (1:1000, AF3500, Affinity Biosciences, Cincinnati, OH, USA). After thorough washing with TBST, the membranes were incubated with a goat anti-rabbit IgG (H + L) secondary antibody (1:5000 dilution, #31460, Thermo Fisher Scientific) for 2 h. Membrane visualization was performed using an Odyssey scanner (Bio-Rad Laboratories, Hercules, CA, USA). Protein band quantification was conducted using NIH Image-Pro Plus v6.0 for relative analysis. Samples were standardized using one sample as the first sample of each gel and the total protein gel as a reference to control for protein loading irregularities [71]. Since internal reference proteins are affected by the origin of the tissue and experimental treatments, they may reduce the accuracy of quantification [72,73,74]. Therefore, we used the method of relative quantification based on total protein.

### 4.9. Statistical Analyses

One liver protein was designated as the standard protein and assigned a value of 1, with the ratios of other groups to this standard representing the relative quantitative results. All statistical analyses were conducted using SPSS v22.0, with data presented as mean ± standard deviation (SD). Overall differences were assessed using a one-way analysis of variance (ANOVA), while group differences were determined using Fisher’s least significant difference (LSD) post hoc test. A *p*-value of less than 0.05 indicated significance.

## Data Availability

The original contributions presented in the study are included in the article; further inquiries can be directed to the corresponding author.
